# Developing senior hospital managers: does ‘one size fit all’? – evidence from the evolving Chinese health system

**DOI:** 10.1186/s12913-020-05116-6

**Published:** 2020-04-06

**Authors:** Zhanming Liang, Peter Howard, Jian Wang, Min Xu, Mei Zhao

**Affiliations:** 1 La Trobe University, Melbourne, Australia; 2 School of Health Care Management (NHC Key Laboratory of Health Economics and Policy Research), Shandong University, Jinan, China; 3 The First Affiliated Hospital of Shandong First Medical University, Jinan, China; 4 University of North Florida, Jacksonville, USA

**Keywords:** Hospital managers, Chinese health system, Management competency, Management training and development

## Abstract

**Background:**

To improve the effectiveness and efficiency of health service provision in China, the National Health Commission has emphasised that training of all health service managers is essential. However, the implementation of that policy has proven challenging for various reasons, one of which is the lack of understanding of the competency requirements and gaps. The aims of the study were to develop an understanding of the characteristics and training experience of hospital managers in one major Chinese city, explore the difficulties they experience and relate them to their perceived importance of management competencies and the perceived level of their management competency.

**Methods:**

A cross-sectional, descriptive study with a three-component survey including the use of a validated management competency assessment tool was conducted with three senior executive groups (*n* = 498) from three categories of hospital in Jinan, Shandong Province, China.

**Results:**

The survey confirmed that formal and informal management training amongst participants before commencing their management positions was inadequate. The core competencies identified in the Australia context were applicable to the management roles in Chinese hospitals. In addition, the senior executives had low levels of confidence in their management competence. Furthermore, the data showed significant differences between hospital categories and management levels in terms of their commitment to formal and informal training and self-perceived management competence.

**Conclusions:**

The study suggests that management training and support should be provided using a systematic approach with specific consideration to hospital types and management levels and positions. Such an approach should include clear competency requirements to guide management position recruitment and performance management.

## Background

Developing the sustainability of healthcare systems to meet growing demands is a global challenge. In the Chinese healthcare system, one of the key threats is the unbalanced distribution of quality and accessibility of medical services [1, 2] and inappropriate and less cost-effective hospital services leading to an escalation in health expenditure [3–5]. Of more concern, this increase has not resulted in improved service provision to patients, and improved outcomes, but an increase in revenue for medical providers and pharmaceutical companies [3–5]. In China, 85% of the health services are provided by public hospitals including inpatient services and outpatient consultations. In 2018, 163.51million admissions and 3.05 billion individual diagnoses and treatment in public hospitals were recorded [6]. This has resulted in overcrowded hospitals and the under-utilisation of community-based clinics and primary care services [1, 2]. There is no doubt that the transformation of the current hospital-centred and fragmented health service delivery system into a more primary care-centred and integrated delivery system in addition to the improvement of quality and efficiency of hospital service provision is required.

Such a transformation would not be successful without competent managers leading and supervising the process. Their importance has been recognised at the central government level. The “Healthy China 2030 program outline” and “The guidelines opinion of building modern hospital management system” published by the Chinese State Council recommend improved competencies for hospital managers [7], stating that hospital development and medical service capacity development requires hospital managers to be more professional in their management skills and their methods/tools used [7].

There is empirical evidence which suggests that management competency is positively linked to improved management outcomes, hence better health service delivery and health service / patient outcomes [8, 9]. Evidence also confirms that competence can be acquired and developed through training and continuous professional development [10, 11]. The increasing investment in formal management training for health service managers in developed countries such as postgraduate programs in health service administration / management is evidence of the importance of management competency development [12]. However, only a small proportion of managers have benefited from the opportunities of formal management training for a number of reasons: i) time and financial constraints, ii) a lack of recognition of management being a professional occupation in the health sector, and iii) a lack of a formal requirement in position descriptions where management qualifications are not broadly recognised as compulsory, even for senior executive positions [13, 14]. Informal training and development with a more short-term and flexible approach plays an undeniable role in management workforce development at individual, organisational and system levels. In the absence of evidence on the relevance and long-term impact of formal education in health service management in terms of meeting the actual demands of the health service management workforce, a better understanding of the current management competency gaps and professional development needs of health service managers is required. Such an understanding would provide evidence to guide the determination of formal, informal and on-the-job training and development directions.

Management in the hospital system varies between management levels and the nature of positions. In addition, the same management level in two hospitals may also differ due to the variable hospital context including size, and the governance and management structure. In the study conducted by Liang et al. (2013a) [15], it differentiates size of hospitals by hospital annual budget and number of beds, and management levels by reporting structures. For example, Chief Executive Officers (CEOs) and Executive Directors (EDs) who report directly to the CEOs are senior managers of a small size hospital when the management level reported directly to EDs are also counted as senior managers in a large size hospital. This classification is applicable to the hospital in many developed countries. However, the management structure is very different in the Chinese hospital system including the recruitment and promotional process for senior executive positions.

In the Chinese hospital system, hospitals are officially and universally classified into 3-tier system of Primary (with less than 100 beds), Secondary (with beds between 100 and 500) or Tertiary (with more than 500 beds) in consideration of a hospital’s geographic location and size [5, 16], and the ability to provide medical care, medical education, conduct medical research [17]. A primary hospital (Level I) is usually a township hospital that provides preventive care, primary health care and rehabilitation services. Secondary hospitals (Level II) are usually located in a medium size city or county that provide comprehensive health services, medical education and conduct research on a regional basis. Tertiary hospitals (Level III) are usually located in major cities that provide comprehensive and specialised health services, play a bigger role in medical education and scientific research, and act as medical hubs providing care to multiple regions [18, 19].

Historically, the Chinese public hospital system is medically dominated system. Vast majority of the senior hospital management positions are filled by clinicians. Therefore, management competencies are developed via hands on experience rather than targeted management training and development. Typically, in a public hospital [20]. the top executive level positions include Executive Directors and Deputy Directors and the Chair and Deputy Chair of the Communist Party. These positions are appointed directly by the Provincial Health Department. There are three types of management positions directly under this top executive level carrying very different management responsibilities (refer to the explanation in the Methods section of paper). The appointments to these positions are usually internal, based on seniority and clinical performance without specific management skills or systematic training requirements [20].

Although the National Health Commission requires all health services managers to receive management training, the implementation of such requirements has proven challenging because of three fundamental issues. Firstly, there are no agreed management standards and requirements currently used to guide hospital managers recruitment other than seniority and clinical performance [21]. Hence, the absence of a management track record [22] and the lack of specific management training among managers prior to taking up management positions is common in public hospitals in China [21]. Secondly, most postgraduate training is non- management and research focused rather than coursework-based / practice-based unlike many Master of Health Administration coursework programs offered by universities in developed countries. This provides very limited postgraduate training opportunities to managers aiming at systematically improving their management competence and their capability in dealing with management related challenges. Thirdly, the absence of requirement of management qualifications means that management related formal training has not been embedded in the job description of management positions. Lastly, actual management competence and management outcomes have not been embedded in regular management performance appraisal providing limited incentives for continuous informal management training and development.

In the Chinese public hospital system, clinicians often take up management roles without a job description with unclear roles and competency requirements. To date, limited empirical evidence has identified the training and competency development needs of managers working in the Chinese healthcare system. A brief search of literature from year 2000 onwards has found only15 studies focussing on the management capacity and competency of managers in Chinese public hospitals [23]. The studies confirmed that less than 7% of hospital managers possessed management related qualifications or receiving extensive management training [22]. These studies reinforce the urgent need to improve management efficiency in Chinese public hospitals by strengthening the management competence of hospital managers in the areas of leadership and decision-making [24].

Although management competencies are context sensitive [25], the literature confirms the existence of core competency requirements across management levels and positions [26–28]. Hence a competency framework identified in one healthcare context can be applied to other contexts after being tested. The existence of core competencies also means that competencies may not carry the same level of importance across positions. The pyramidal relationship between tasks, competencies, knowledge, skills and attitude as explained in Liang et al. (2013b) [29] confirmed that competency requirements may vary between management positions. Therefore, an understanding of the differences will provide evidence to shape the design of management training and development, both informally and formally, for health service managers in specific healthcare context and positions.

It is in this context, a large-scale survey was conducted in three hospitals located in Jinan, the capital city of Shandong Province located in the northern part of China. One objective of the study was to develop an understanding of the three most senior hospital management positions in terms of their work experience, educational background and training received before and after taking up the management position. The second objective aimed to understand the perceived importance of management competencies to management roles, the difficulties encountered and the perceived level of their management competency. Based on the findings, the paper will discuss how formal education, management positions and hospital category effect the difficulties encountered in management roles and the perceived importance of management competencies and perceived level of management competence.

## Methods

This study was cross-sectional and descriptive in nature.

### Target population

The target population included the following management categories from three hospitals in Jinan, Shandong Province, eastern China. These three hospitals represent the 3-tier system of hospital categorisation. Jinan Qian FoShan Hospital (QFSH) is a Level III hospital located in Jinan, the capital city of Shandong Province. Lai Cheng Qu Hospital (LCQH) is a Level II hospital located in a suburb of Jinan, Xi Xian Hospital (XXH) is a Level I hospital located in the county area in Shandong Province.

1. Executive Directors and Chair and Deputy Chair of Communist Party (ED)
2. Heads of the Administration and Functional Departments (HoA)
  - Directors of Administration and Functional Departments (DoA)
  - Associate Directors of Administration and Functional Departments (ADoA)
3. Heads of the Clinical Services (HoCS)
  - Directors of Clinical Services (DoCS)
  - Associate Directors of Clinical Services (ADoCS)
4. Directors of Nursing (DoN)

For the purpose of this paper, the Executive Directors were excluded from the analyses as they were a very small percentage of the total target population and regarded as a special group in terms of roles and responsibilities.

### Questionnaire

A survey was conducted with potential participants in the targeted management positions from three hospitals in Jinan City. The questionnaire was developed in English and then translated into Mandarin. To maximise accuracy, it was then back translated into English by an independent collaborator and the Mandarin version revised for clarification where necessary. Each questionnaire took approximately 20 min to complete and consisted of three components:

1. Demography, educational background, and previous and current work experience;
2. Past and current training, specifically management related, and perceived management difficulties, and.
3. Perceived importance and self-assessment of competence for the six core management competencies using the validated MCAP management competency tool [30], which were:
  C1. Evidence-informed decision-making *(Evidence)* – 13 behavioural items
  C2. Operations, administration and resource management *(Resources)* – 17 behavioural items
  C3. Demonstrated knowledge of healthcare environment and the organisation *(Knowledge)* – 11 behavioural items
  C4. Interpersonal, communication qualities and relationship management *(Communications)* – 19 behavioural items
  C5. Leading people and organisations *(Leadership)* – 13 behavioural items
  C6. Enabling and managing change *(Change)* – 9 behavioural items

The validated MCAP 7-point descriptive scale [30] (Additional file 1: Appendix 1) was used for participants to assess their own competency level. Participants were also asked to self-assess their level of competence for the 82 behavioural items for the six competencies. The results of behavioural items self-assessment will be the focus of another paper.

The link accessing the online questionnaire using the Qualtrics survey platform https://www.qualtrics.com/ was distributed by one of the QFSH Deputy Executive Director (DED) directly to the targeted management positions at each of the three hospitals and was open for a two-week period between Nov 23rd and Dec 6th, 2018. Three reminders were sent from the DED to all potential participants during this two-week period. Due to the low response rate amongst HoCS at QFSH and after discussions with QFSH, a paper-based survey with the same content as the online version was distributed in February 2019 to these positions to encourage higher response rate. Completed paper-based survey was collected within 2 weeks after the distribution.

### Data management and analysis

The data were downloaded from the Qualtrics website into MS Excel format. In addition, the data from the paper-based questionnaires were entered into MS Excel. The two datasets were merged. Following error checking, the means of the six competencies and the combined competencies were calculated. All data were then imported into IBM SPSS ver. 25 for analysis.

For ease of analysis, three summary scores were calculated. The first score was a summary of the number of different topics of management training experienced before the participants took up their management roles. The second score summarised the number of management topics taken up by participants during their management positions. The third score enumerated the number of difficulties that the participants experienced in their current position.

Univariate analyses were carried out for all variables and separately by hospital and management level. Differences between management levels and / or hospital were tested for statistical significance by crosstabulation and chi square tests or by univariate analyses of variance.

## Results

In total, 513 managers participated in the survey including 15 EDs, 62 HoA, 295 HoCS, and 141 DoNs from the three targeted hospitals. Table 1 provides details of the target population and response rates for each management level by hospital. With the exception of one subgroup (the HoA at XXH), the response rates were satisfactory.

**Table 1:** Target population and response rates by hospital and management level

		ED	HoA	HoCS	DoN	Total
**QFSH**	Total No.	7	38	212	104	361
	Participants	4	30	209	103	346
	Response rates	57%	79%	99%	99%	96%
**LCQH**	Total No.	6	24	47	25	102
	Participants	4	21	42	25	92
	Response rates	67%	88%	89%	100%	90%
**XXH**	Total No.	8	24	44	14	90
	Participants	7	11	44	13	75
	Response rates	88%	46%	100%	93%	84%
**Total**	Participants	15	62	295	141	513

*QFSH* Jinan Qian FoShan Hospital, *LCQH* Li Cheng Qu Hospital, *XXH* Xi Xian Hospital

*ED* Executive Directors, *HoA* Head of Administration and Functional Departments, *HoCS* Head of Clinical Services, *DoN* Directors of Nursing

### Demography and employment details

The gender ratios varied by management level from 1.8:1 for clinical managers to 0.14:1 for nursing managers. Overall, the mean age of participants of the three management groups ranged from 40.6 to 47.2 years. DoN tended to be younger than all other management positions. Managers generally spent about 13 years working in non-management roles before advancing to management positions. HoCS spent approximately 2 years longer than managers in other positions before taking up management role. Table 2 details the above differences between positions.

**Table 2:** Gender, age and years in non-management and management roles by management level

Items	HoA	HoCS	DoN	Total
**Male:Female ratio**	0.68:1	1.8:1	0.14:1	0.77:1
**Mean Age (years)**	44.8	47.2	40.6	45.0
**Mean total years working in non-management related roles**	11.3	13.7	12.0	12.9
**Mean total years working in hospital system**	21.6	23.6	19.6	22.2
**Mean number of years in the current management position**	4.4	6.7	5.4	6.1
**Mean total years working as a manager**	10.3	9.9	7.7	9.3

*HoA* Head of Administration and Functional Departments, *HoCS* Head of Clinical Services, *DoN* Directors of Nursing

### Postgraduate qualifications

Less than half (44%) of the participants possessed a postgraduate qualification. Of these 56% were PhDs and 44% master’s degrees (Table 3). Clinical directors had the highest percentage of postgraduate qualifications (57%) with 94% of PhDs. Directors of Nursing had the lowest percentage of postgraduate qualifications (21%). The clear majority of the postgraduate degrees (98%) were held by managers at QFSH. None of the managers from XXH and LCQH and none of the Directors of Nursing at QFSH had acquired a PhD. Only 13 out of the 96 Master’s Degrees (14%) and three out of 122 PhDs (2.5%) were management related. Most of degrees were in the discipline of medical science.

**Table 3:** Postgraduate Qualifications (Masters or PhD) by management level and hospital

	HoA	HoCS	DoN	Total
**Number of managers with postgraduate qualification**	20	168	30	218
**Percentage of managers with postgraduate qualifications**	32%	57%	21%	44%
**QFSH**	19 (63%)	164 (79%)	20 (29%)	213 (63%)
**LCQH**	1 (5%)	4 (10%)	0 (0%)	5 (6%)
**XXH**	0 (0%)	0 (0%)	0 (0%)	0 (0%)
**Postgraduate degrees in management related discipline**	6 (30%)	9 (5%)	1 (3%)	16 (7%)
**Managers with PhD (QFSH only)**	7 (23%)	115 (55%)	0 (0%)	122 (36%)

*QFSH* Jinan Qian FoShan Hospital, *LCQH* Li Cheng Qu Hospital, *XXH* Xi Xian Hospital

*HoA* Head of Administration and Functional Departments, *HoCS* Head of Clinical Services, *DoN* Directors of Nursing

In addition, 28 directors were currently completing either a Master’s degree or PhD. However, only three of these degrees were management related (Table 4).

**Table 4:** Number of directors currently completing a postgraduate qualification by hospital and management level

	HoA	HoCS	DoN	Total
**QFSH**	4	10	9	23
**LCQH**	0	1	1	2
**XXH**	0	2	1	3
**Management related discipline**	2	1	0	3

*QFSH* Jinan Qian FoShan Hospital, *LCQH* Li Cheng Qu Hospital, *XXH* Xi Xian Hospital

*HoA* Head of Administration and Functional Departments, *HoCS* Head of Clinical Services, *DoN* Directors of Nursing

### Informal management related training

Overall, between 50 and 64% of the managers from three different management positions participated in some form of management related training before taking up their current management positions. The rates varied by management level and hospital. The participation rate increased to 66–79% after taking up the management role (Table 5). The participation rates of HoCS were slightly lower than other management levels.

**Table 5:** Percentage of managers participating in management related training by hospital and management level

		HoA	HoCS	DoN
**Before taking up current management position**	QFSH	57%	54%	63%
	LCQH	62%	37%	64%
	XXH	64%	48%	69%
	Total	60%	50%	64%
**After taking up current management position**	QFSH	72%	67%	85%
	LCQH	81%	51%	68%
	XXH	64%	77%	62%
	Total	74%	66%	79%

QFSH: Jinan Qian FoShan Hospital; LCQH: Li Cheng Qu Hospital; XXH: Xi Xian Hospital

*HoA* Head of Administration and Functional Departments, *HoCS* Head of Clinical Services, *DoN* Directors of Nursing

DoNs consistently had the highest participation rates before and after taking up their management roles. Looking at the data separately by hospital and management level (Table 5), less than 50% of the HoCS at LCQH and XXH took part in management related training before taking up the management roles; this represents the lowest participant rate amongst all management positions across the three hospitals. However, the participation rate for these managers increased by more than 14% after taking up the management position. HoA at LCQH and DoN at QFSH had the highest participation rate of slightly more than 80% after taking up the management position. Overall, the participation in management related training was consistently higher after taking up the management role compared to before across management positions and hospitals.

### Management training topics

**Fig. 1:**
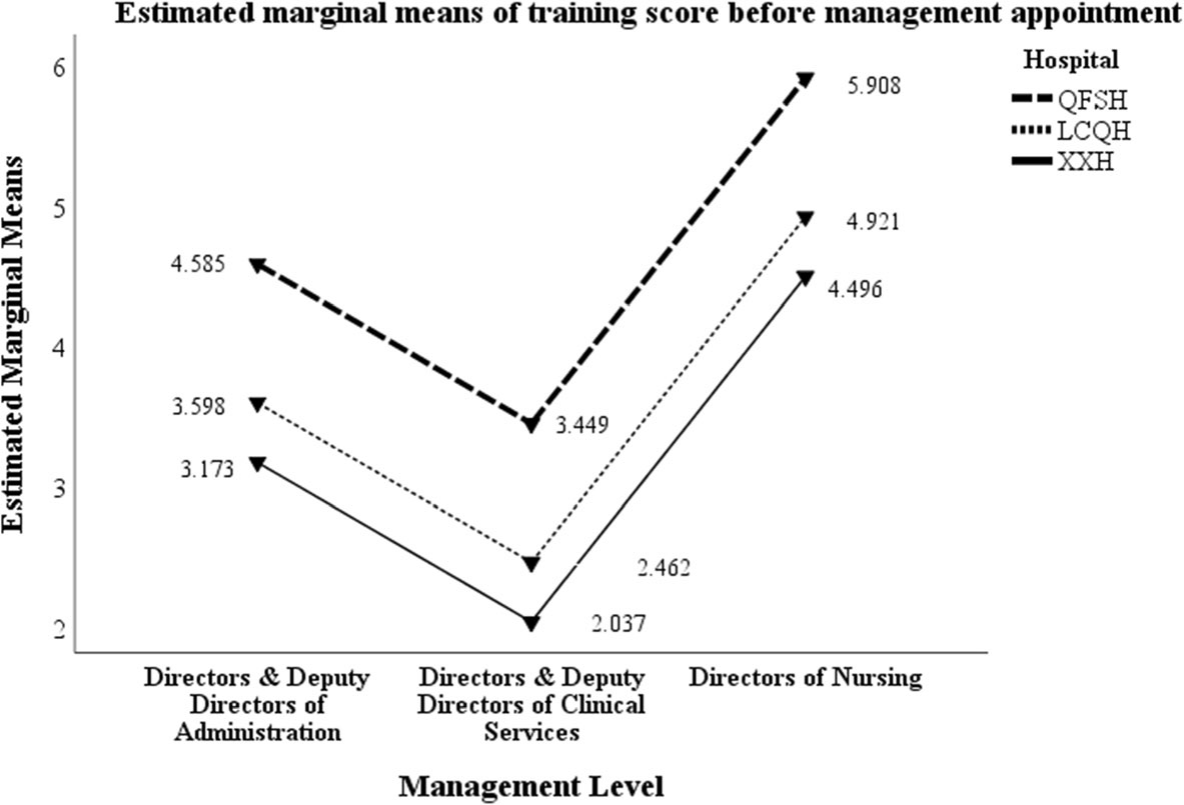
Mean training scores before current management appointment by hospital and management level

**Fig. 2:**
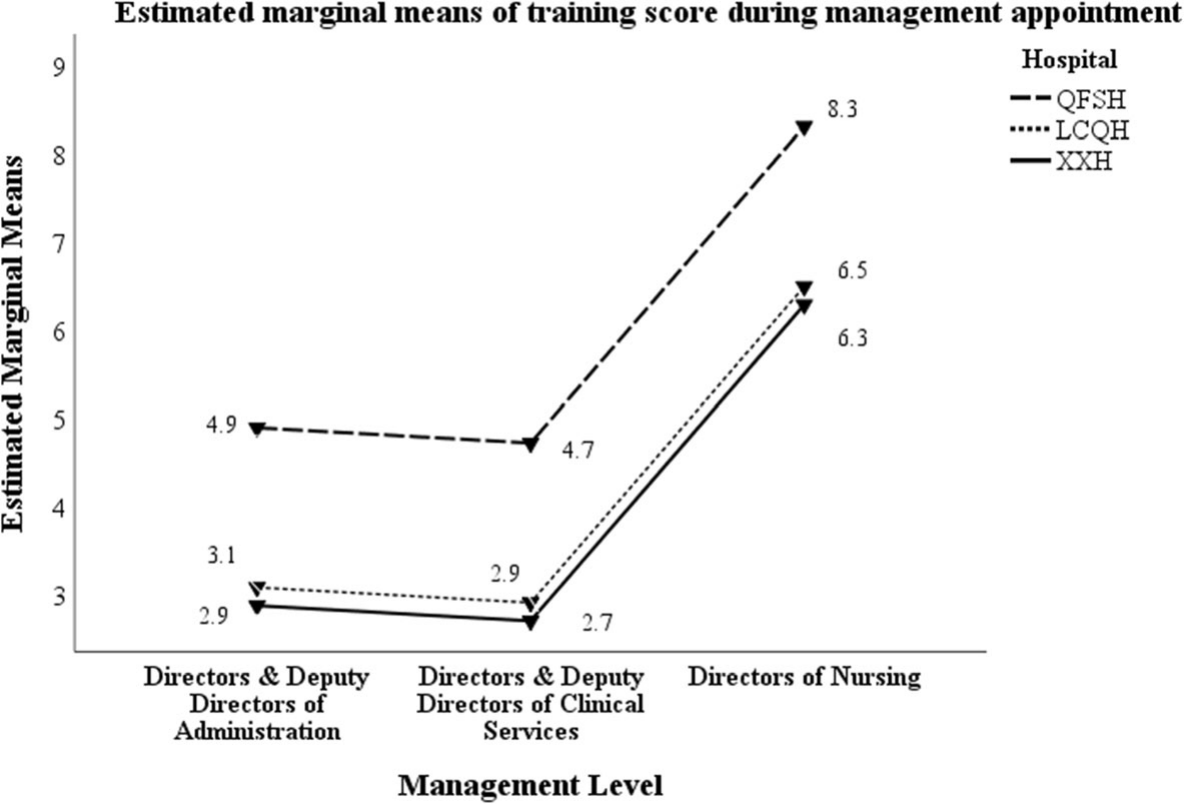
Mean training scores during current management appointment by hospital and management level

In total, 16 topics of management related training were provided to participants for multiple selection. Table 6 details the mean scores for training before and during current management role by hospital and management level. Managers at QFSH attended significantly more management training in terms of types both before and during taking up their management positions compared to the other two hospitals. Directors of nursing attended significantly more training than the other types of directors. Figures 1 and 2 represent the data graphically.

**Table 6:** Mean scores for training before and during current management role by hospital and management level

Hospital and Management Level		Mean score before	Mean score during	N
**QFSH**	HoA	5.17	4.60	30
	HoCS	3.28	4.44	209
	DoN	6.08	8.96	103
	Total	4.29^**a**^	5.81^**c**^	342
**LCQH**	HoA	2.71	3.48	21
	HoCS	3.14	3.52	42
	DoN	4.52	5.12	25
	Total	3.43^**a**^	3.97^**c**^	88
**XXH**	HoA	3.27	2.91	11
	HoCS	2.18	3.45	44
	DoN	3.92	3.69	13
	Total	2.69^**a**^	3.41^**c**^	68
**Total**	HoA	4.00^**b**^	3.92^**d**^	62
	CS	3.10^**b**^	4.16^**d**^	295
	DoN	5.60^**b**^	7.79^**d**^	141
	Total	3.92^**a,b**^	5.16^**c,d**^	498

*QFSH* Jinan Qian FoShan Hospital, *LCQH* Li Cheng Qu Hospital, *XXH* Xi Xian Hospital

*HoA* Head of Administration and Functional Departments, *HoCS* Head of Clinical Services, *DoN* Directors of Nursing

^a^Hospital: Mean Square = 75.293; F = 3.269; *p* = 0.039

^b^Management level: Mean Square = 289.563; F = 12.574; *p* < 0.0001

^c^Hospital: Mean Square = 189.57; F = 7.91; *p* < 0.0005

^d^Management level: Mean Square = 634.696; F = 26.481; *p* < 0.0001

Additional file 1: Appendices 2a and 2b present the percentage of managers completing the training topics before and after taking up their current management positions, by management level and hospital. Managers at QFSH completed more training types both before and during their current management positions compared to the other two hospitals. HoCS completed significantly less management related training before taking up their management roles. In contrast, DoNs completed significantly more types of management related training both before and after taking up the current management position than all other management positions. Across all hospitals and management levels, more training was completed after taking up the management roles compared with before.

Of all the management training areas, conflict resolution, employee relationships, safety training, performance management, leadership, human resource management and communications were the seven areas which attracted the highest participation (26–37%) across all management positions before taking up the management positions. After taking up their management roles, an additional five topics (time management, decision-making, resource management, quality control and policy & procedure) also attracted higher participation rates (27–35%).

### Commitment to training and professional development

Participants were also asked to recall whether they had participated in any of the training as listed in Table 7 for more than 10 h per year in the past 3 years. The table indicates the percentage of managers participated in the types of training for no less than 10 h per year in the last 3 years are included in the table. Overall, 71% of all managers participated in management related training organised internally and 42% of all managers participated in management related training externally for more than 10 h annually. However, less than half of the managers from each type of management positions committed more than 10 h annually in self-study on management-related topics. The participation rate for HoCS was only 22%.

**Table 7:** Percentage of managers committing to training and professional development by management level

	HoA	HoCS	DoN	Total
Non-management related training organised or provided by the current hospital	27.4%	54.6%	54.6%	52.2%
Management related training organised or provided by the current hospital	59.7%	69.8%	74.5%	70.6%
Non-management related training organised or provided externally	66.1%	48.1%	73.0%	57.3%
Management related training organised or provided externally	53.2%	39.3%	40.4%	41.9%
Self-study on management-related topics	38.7%	21.7%	34.8%	28.1%

*HoA* Head of Administration and Functional Departments, *HoCS* Head of Clinical Services, *DoN* Directors of Nursing

### Difficulties encountered in the management position

**Fig. 3:**
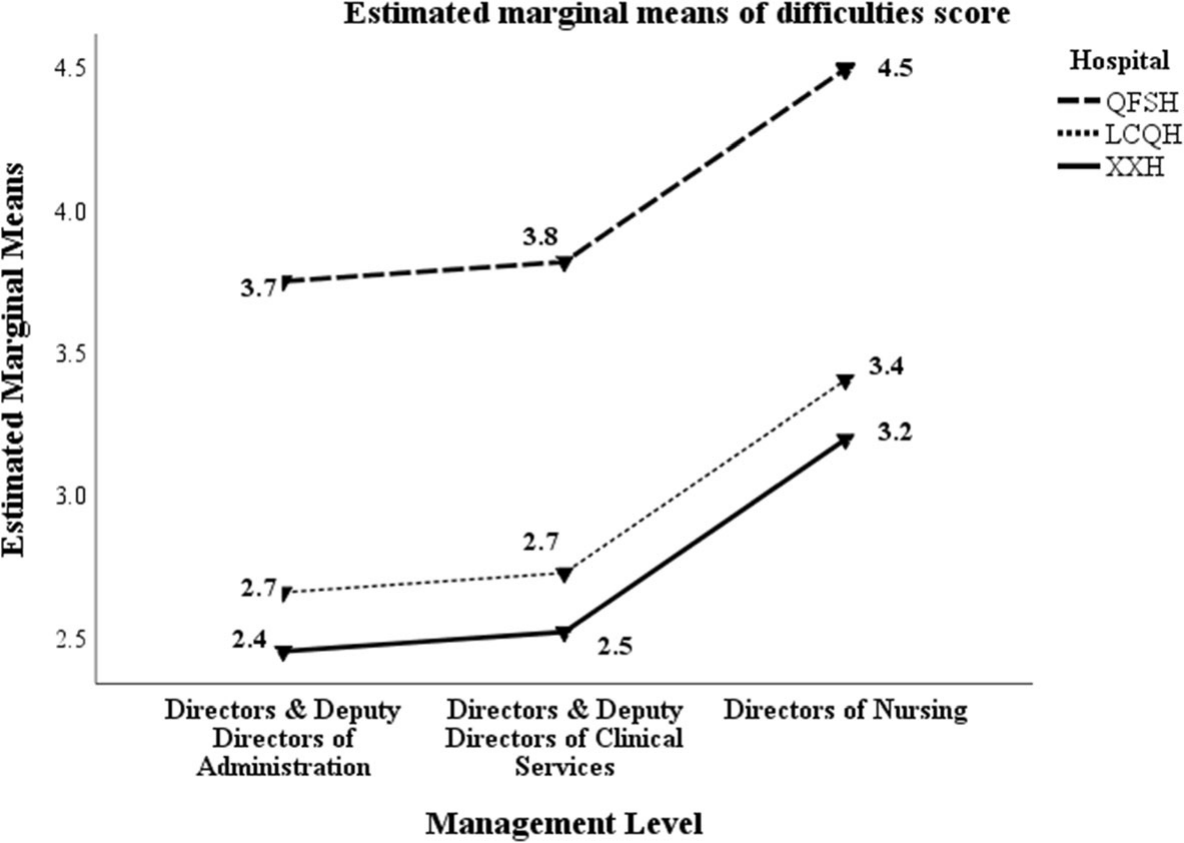
Mean difficulty scores by hospital and management level

Participants were also asked to indicate the difficulties encountered while in their current management position. A list of 15 difficulties were provided for multiple selection. Table 8 shows the mean difficulties scores by hospital and management level. The scores of those selected by QFSH managers are significantly higher than the other two hospitals. In addition, the scores of the directors of nursing are significantly higher than the other management positions (refer to Fig. 3).

**Table 8:** Mean difficulty scores by hospital and management level

Hospital and Management Level		Mean difficulty score	N
**QFSH**	HoA	3.80	30
	HoCS	3.67	209
	DoN	4.76	103
	Total	4.01^**a**^	342
**LCQH**	HoA	3.05	21
	HoCS	3.26	42
	DoN	2.16	25
	Total	2.90^a^	88
**XXH**	HoA	1.55	11
	HoCS	2.66	44
	DoN	3.46	13
	Total	2.63^**a**^	68
**Total**	HoA	3.15^**b**^	62
	HoCS	3.46^**b**^	295
	DoN	4.18^**b**^	141
	Total	3.63^a**,**b^	498

*QFSH* Jinan Qian FoShan Hospital, *LCQH* Li Cheng Qu Hospital, *XXH* Xi Xian Hospital

*HoA* Head of Administration and Functional Departments, *HoCS* Head of Clinical Services, *DoN* Directors of Nursing

^a^Hospital: Mean Square = 73.815; F = 10.257; *p* < 0.00005

^b^Management level: Mean Square = 23.918; F = 3.323; *p* = 0.037

Additional file 1: Appendix 3 shows the percentage of managers selecting the difficulties by management level and hospital. Except for HoAs at QFSH, patient conflict was the commonest difficulty selected by the directors (36–62%). Other commonly selected difficulties (greater than 25%) included peer conflict, team conflict, innovative teamwork, employee performance, decision-making, new skill acquisition, expected work quality and management outcomes expectations. There was considerable variation between hospitals and management levels; managers at QFSH tended to report more difficulties than the other hospitals.

### Perceived importance and self-assessment of management competencies

All participants were asked to indicate the importance of each of the six core management competencies to their current management role and whether they had acquired these competencies prior to taking up the current management position. Using a 5-point Likert importance scale, the vast majority of the managers (ranging from 84 to 98%) perceived the six competencies as important or very important to their management role. Less than 3.5% of all managers indicated any of the six competencies being unimportant or very unimportant (data not shown).

Another 5-point Likert scale was used to ask managers to indicate the extent to which the competencies had been acquired before taking up their current management role. Table 9 details the percentage of directors identifying the extent that they had acquired each of the competencies before taking up their current management roles.

**Table 9:** Percentage of managers acquiring competencies before taking up their current management position

Competency	Not at all	Acquired to limited degree	Unsure	Cumulative percentage	Acquired most of it	Fully acquired
**C1. Evidence**	2.3	11.4	15.6	29.3	56.1	14.6
**C2. Resources**	5.0	13.5	22.5	41.0	48.2	10.8
**C3. Knowledge**	0.2	7.5	10.0	17.7	60.7	21.6
**C4. Communications**	0.4	5.6	8.3	14.3	61.5	24.1
**C5. Leadership**	3.3	10.4	18.3	32.0	53.2	14.8
**C6. Change**	6.7	13.7	23.5	43.9	42.8	13.3

Table 9 indicates that, for each of the six competencies, 11 to 24% of all directors perceived themselves as having fully acquired the competencies. More managers acquired competencies 3 and 4 (21.6% for Knowledge and 24.1% for Communications) than competencies 2 and 6 (10.8% for Resources and 13.3% for Change). Conversely, between 14 to 44% of all directors indicated that they had not acquired, only acquired to a limited degree or were unsure for all competencies, the highest were for competencies 2 and 6 (41% for Resources and 43.9% for Change).

### Competency level – self-assessment

According to the description of MCAP Likert scale (Additional file 1: appendix 1), a competency score of five (5.0) or greater indicates that participants could demonstrate the competency in their role independently without guidance. Table 10 provides details of the mean scores for the six competencies and by management level.

**Table 10:** Means of self-perceived management competencies by management level

	DoA / ADoA	DoCS / ADoCS	DoN	Total
**C1. Evidence**	3.37	4.33	3.81	4.07
**C2. Resources**	3.22	4.23	3.66	3.94
**C3. Knowledge**	3.78	4.59	4.22	4.39
**C4. Communications**	3.87	4.70	4.45	4.53
**C5. Leadership**	3.65	4.40	3.94	4.17
**C6. Change**	3.32	4.14	3.52	3.86

*HoA* Head of Administration and Functional Departments, *HoCS* Head of Clinical Services, *DoN* Directors of Nursing

**Fig. 4:**
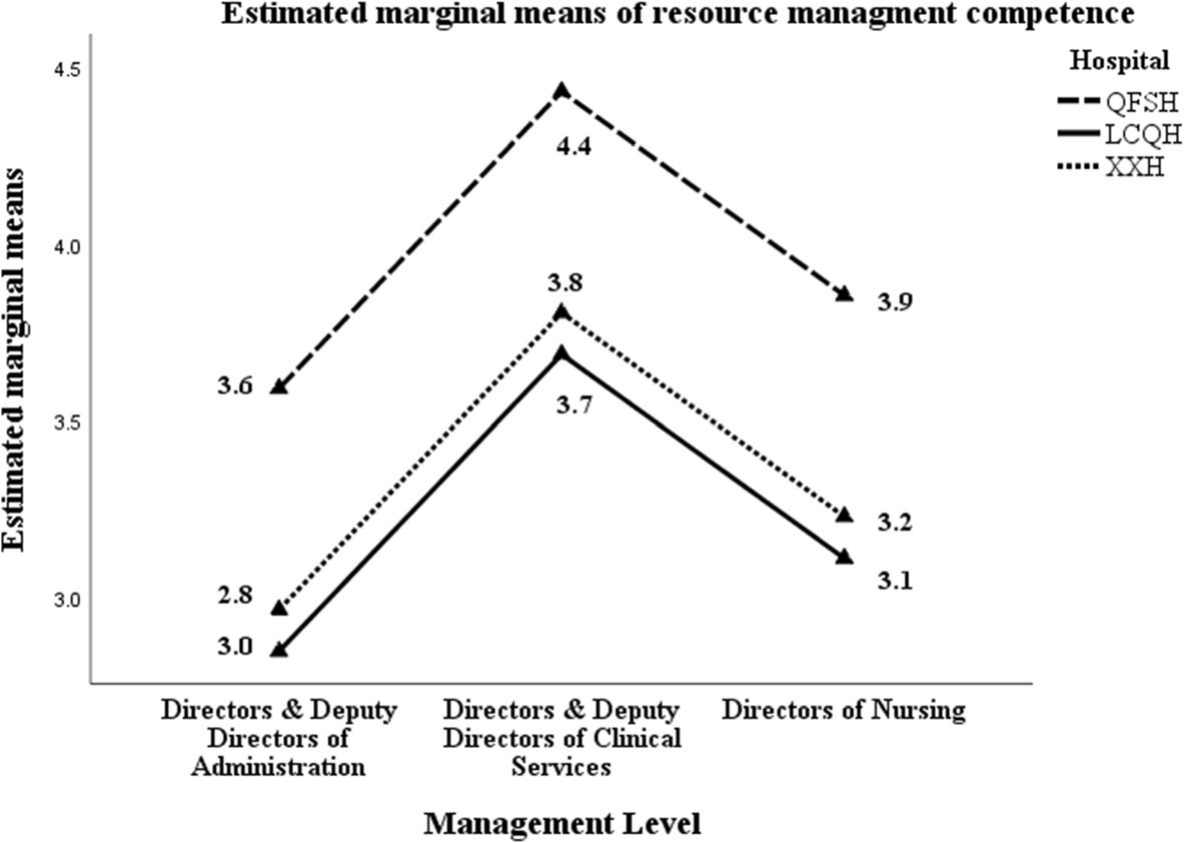
Means of competency 2 (resource management) by hospital and management level

All six competencies were scored less than five for all managers levels. Competencies 2 & 6 were scored less than four overall (‘*fully demonstrate in my role but with regular guidance*’). Examining the scores by the three management levels, HoA recorded scores less than four (means ranging from 3.22 to 3.87) for the six competencies. DoNs also recorded mean scores lower than four for competencies 1, 2, 5 & 6. Mean scores for competencies for HoCS ranged between 4.14 and 4.70. Competency mean scores for HoCS were all higher than HoA and DoN, and the differences between the management levels were all highly significant as measured by ANOVA (F = 8.532–14.862; *p* - < 0.00005). Figure 4 is typical of all the competencies.

If the hospital variable is included as a predictor in the univariate analysis of variance model, there are significant differences between hospitals with managers at QFSH assessing themselves significantly higher than managers at the other two hospitals (Mean Square = 23.857; F = 11.403; *p* < 0.00005). Figure 4 is typical of all the competencies.

Other statistically significant predictors of the self-perceived competency levels in a bivariate relationship included sex (M > F), age (positive correlation), total number of years as a manager (positive correlation) and postgraduate qualification (PG > UG). However, when added to a univariate model with the hospital and management level variables, both sex and the number of years as a manager ceased to have a significant effect on all competencies. Age remained a significant predictor for all six competencies and the combined competency. Manager type remained a significant predictor for all competencies except C5. Postgraduate education also remained a significant predictor for competencies C3, C4, C5, C6 and combined competencies. Hospital level remained significantly significant for competencies C1, C4, C5 and the combined competencies. The model included an interaction term of age times number of years as a manager, as these two variables were moderately highly correlated. Table 11 shows the results of the univariate model for C4 (Communications).

**Table 11:** Results from a univariate model for C4 (Communications)

Source	Type III Sum of Squares	df	Mean Square	F	Sig.	Partial Eta Squared
**Corrected Model**	180.005^a^	9	20.001	11.756	0.000	0.187
**Intercept**	4.529	1	4.529	2.662	0.103	0.006
**Hospital**	16.745	2	8.373	4.921	**0.008**	0.021
**Management Level**	25.938	2	12.969	7.623	**0.001**	0.032
**Sex**	0.629	1	0.629	0.370	0.543	0.001
**Postgraduate**	6.343	1	6.343	3.728	**0.045**	0.008
**Age**	31.360	1	31.360	18.432	**0.000**	0.039
**Total years as manager**	2.635	1	2.635	1.549	0.214	0.003
Age * Total years as manager	1.216	1	1.216	0.715	0.398	0.002
**Error**	780.912	459	1.701			
**Total**	10,571.000	469				
**Corrected Total**	960.917	468				

^a^. R Squared = 0.187 (Adjusted R Squared = 0.171)

## Discussion

A very high proportion of the targeted population participated in the survey with an overall response rate of approximately 90% and response rates ranged between 72 and 94% amongst the four different positions. Such a high response rate was partly attributed by the commitment from the participating hospitals, especially the encouragement provided by the Executive Directors.

The study found significant differences between different management positions and the same positions across the three hospital categories in several areas in terms of postgraduate qualifications, commitment to informal management training, difficulties encountered in the management roles and the self-perceived management competency levels. In general, managers in the category III hospital had higher levels of education and higher exposure to management related training than their colleagues in the smaller category II and I hospitals. They also had higher levels of self-perceived management competence across the six core management competencies. However, they also faced more challenges/difficulties in their management positions. The differences indicate that strategies to develop and support managers in three hospital categories vary.

### High education level versus low participation of management training

On average, the HoCS were older than the HoA and DoN. This group also had a much larger proportion (57%) with postgraduate qualifications including both Masters and PhDs. In addition, nearly 64% of HoCS at QFSH had a PhD, a high rate which was rarely found among senior managers in other studies; however, the majority of the degrees were not management related. This high rate did not apply to the same management positions in the other two hospitals, which are in a lower hospital category. This indicates higher qualification requirements for the positions of HoCS in the Level III hospital. Whether this is a unique requirement for QFSH only or common to other level III hospitals in China needs further investigation.

Nevertheless, other studies have found similar trends. For example, a project investigating the Australian sectors that employ PhD holders and the career ambitions of current PhD students found that the public and private health and medical sectors employ the largest number of PhDs [31]. In the public sector, the main area of PhD employment is health care especially hospitals. However, the number of these PhD holders who were in the hospital leadership positions was not mentioned in the project report. Similarly, the United States Bureau of Labor Statistics found that the employment of medical and health services managers is projected to grow 18% from 2018 to 2028, much faster than the average for all occupations. Correspondingly, the demand for postgraduate degrees in this discipline will grow during the next decade [32].

The possession of high qualifications such as doctorate degrees does not mean that the participating managers have acquired adequate management training because less than 5% of all postgraduate qualifications possessed by participating managers were management related. This is slightly lower than the 7% identified in recent studies conducted in other parts of China [23]. Although more than 50% of managers participated in some forms of informal management related training either organised internally by hospitals, or provided externally, or via self-study, the participation rate in various types of management training is generally lower than 50%. Less than half of the HoA and HoCS participated in the management training types either prior to or after taking up their management positions.

The findings contrast to a recent management competency study in Australia targeting senior managers in both public hospitals and community health services [26]. This study confirmed that more than two thirds of the senior managers working in both the public hospitals and community health services in Victoria (one of the largest Australian States) possessed a postgraduate qualification. Amongst the postgraduate qualifications that Australian health service managers possessed, one third of them were management related. The results of the present study confirm that the advancement to a management career in China is based on seniority in clinical practice rather than a management related background providing limited motivation for potential candidates of management positions in developing management competence prior to taken up management roles. This may explain the increased level of commitment in management related training after taking up the management roles amongst the study participants.

However, such motivation may not be high enough to encourage managers to commit time and energy to continuous improvement of management competence. Nearly one third of the managers spent less than 10 h in management related training annually and less than half of the manager committed more than 10 h annually in self-study on management-related topics. The commitments were much lower amongst the HoCS. This may be partly due to the fact that management specific training has not yet become a requirement for management roles in the healthcare sector in China and the lack of recognition of formal and informal management training [7, 20]. This is compounded by the difficulties for managers to take time off to commit to fulltime or part-time study. However, their perception of the importance of training to improve their management competence and thus management outcomes has yet to be investigated.

As mentioned in the introduction, training and education in health service management / health administration has received greater support among developed countries [33, 34] and effective training is an important human resource management practice for improving the competence of the health service management workforce [10, 11]. Such recognition is partly due to the generation of evidence linking the competence of health managers with better health service delivery and linking the improved competence of health managers via formal management training and education [8, 9, 33]. The overall lack of management related training received by senior hospital managers in Jinan indicates that a combination of individual, organisation and system wide approaches are required. The simple investment in management training by individual managers is far from adequate to improve the competence of the health service management workforce.

### Management competency self-perception – importance, acquisition and competence

Vast majority of the managers (more than 90%) confirmed the importance of the six core competencies in the MCAP framework to their management position. Less than 4% of managers indicated any of the six competencies being unimportant or very unimportant. This confirms the ‘existence of core management competencies across management levels and position theory’ [27, 28, 35]. Ignoring their importance, not all managers believed that they had acquired the required competencies prior to taking up the current management positions. Close to 20% of all managers indicated that they had not acquired or acquired to a limited degree of the competencies 2 (Resources) and 6 (Change). Amongst the three management positions, HoA and DoN are less prepared than HoCS. Close to 20% of HoA also did not acquire or acquire to a limited degree of competencies 1 (Evidence) and 5 (Leadership).

The confirmation of the inadequate acquisition of competency C2 and C6 prior to taking up the current management positions are confirmed by the low levels of self-perceived competence when managers were asked to rate their competency level against each management competency using the validated MCAP competency scale [27]. Overall, C2 and C6 were the only two competencies which received mean competency scores lower than four amongst all managers. A similar study conducted with senior managers in public hospitals and community health services in Australia also confirmed C2 and C6 were two of the competencies in which managers were least confident by self-competency assessment [35].

As mentioned in the results section, none of the six competencies received a mean score higher than 4.7 by any of the management positions indicating that majority of the senior managers cannot demonstrate the required competencies independently without occasional guidance. HoAs recorded means of less than four (between 3.22 and 3.87) for all of the competencies, and DoNs also recorded mean scores lower than four for competencies 1, 2, 5 & 6 and mean score of 4.22 and 4.55 for C3 and C3 respectively. Mean scores received from HoCS for six competencies ranged between 4.14 and 4.70. Mean competency scores for HoCS were significantly higher than the other two positions.

All participants of the study were managers taking on the most senior leadership and management responsibilities in functioning areas or clinical wards, hence high management competence level with a score of five or higher were expected [30]. However, the results of the self-perceived level of competence did not reflect the levels expected of their management positions. Although self-assessment of one’s own competency may provide subjective information, the consistent low scores at least indicate the lack of confidence in the management capability amongst these top-level managers in the three types of public hospitals. On the other hand, the value of self-assessment has been confirmed as an important step of performance management [36]. Research evidence indicates that the self-judgement of one’s own skills and abilities positively relates to self-ability to learn which can improve competence [33] - another important phase of performance management. Hence, the self-assessment of own competence amongst senior managers may assist hospitals toward developing effective HRM strategies and performance management.

### Difficulties encountered

This study found that “conflicts with patients”, “confronting an employee performance problem”, and “having to learn something new such as information or medical technology” are the three difficulties encountered by most of these leadership groups in all three hospitals. The findings are consistent with the well published hospital adverse events and tense patient-doctor relationships in recent years in China. This may be specific to the existing social and health system context specific to China. Studies overseas, especially in developed countries, the well-published biggest challenges to senior managers are more on quality and safety related issues rather than issues of conflicts with patients and communication [37]. However, the study identified that the difficulties facing Chinese senior hospital managers are consistent with managers in other countries, including inadequate employee performance evaluation systems [37].

The advancement of medical technology has changed the way how providers practice medicine today and will continue to do so in the foreseeable future. Technology has improved the effectiveness of diagnosis and treatment but requires new skills to understand how to use it and the information generated. In addition, health organizations nowadays are more geographically widespread requiring “low-cost alternatives to office visits” and in-patient care [38]. The emergence of eHealth and telemedicine allows such transition to take place. The rapid changes require leaders to acquire and develop competencies in this area to manage and store the information adequately. In addition, these leaders can prepare continuous training strategies for the staff and the patient to keep up with the continual change of technology and medicine in the future.

### Management competency predictors

Self-assessed management competency in this study varied significantly by hospital category and management level. The study found that managers from the category III hospital (large size, university affiliated teaching hospital) have higher levels of confidence in their management competence even though they also encounter more difficulties in their current management roles compared to managers from the two smaller hospitals. With the affiliation with a university, a larger patient base and better infrastructure, Category III hospitals may be in a better position to attract better qualified clinicians graduating from leading medical schools. On the other hand, the pressures they face would require more targeted training and support provided by the hospitals to not only further improve their management competency, but also to prevent burn out in consideration of the fact that category III hospitals often attract more patients, hence heavier workloads.

In consideration of the above findings, it is reasonable to make the following three suggestions. Firstly, system wide strategies are required to develop a competent management workforce for the Chinese health system, particularly the recognition of the important roles of health service managers, establishing clear competency requirements for management positions to guide recruitment and performance appraisal. Secondly, specific support structures with incentives should be developed by each hospital to encourage the uptake of formal and informal management related training. Thirdly, developing a mechanism allowing cross management position learning, specially allowing junior and emerging managers to be coached by experienced leaders thus forming succession planning strategies. Fourthly, given the Chinese government direction of health service management workforce development and successful overseas experience, committing to formal and informal management related training is a critical step for existing managers to gain confidence and competence in their management roles, and an important preparation for those who would like to advance their clinical career into a dual role of management.

### Strengths and weaknesses

The major strength of the study is the sample size and high response rates across hospitals and management levels. One weakness of the study was the reliance on self-reported information which may challenge its internal validity. In addition, only one hospital per category was used in the study, which may limit the external validity of the results.

## Conclusions

Targeting senior managers from hospitals representing three different Chinse hospital categories in the capital city of Shandong Province in China, the study not only confirms the importance of the six core competencies identified in an overseas context to their management roles, but also the lack of confidence of these managers in demonstrating these competencies. The low level of confidence is more significant amongst the managers from the smaller and less resourced category II and I hospitals and also amongst the Directors and Associate Directors of Administration and Directors of Nursing who have a significantly lower levels of postgraduate qualification. The study also confirms the lack of investment in management related training in both formal and informal formats prior to and after taking up management positions indicating the lack of readiness and preparation of senior managers. Support structures should be developed by each hospital to encourage the uptake of formal and informal management related training. The study suggests a systematic approach to develop the hospital senior management workforce in China including providing recognition and incentives, establishing clear management competency requirements to guide management position recruitment, development and performance management.

## Supplementary information

**Additional file 1: Appendix 1.** MCAP Competency Likert Scale. **Appendix 2.** a**)** Percentage of managers completing training topics before taking up the current management position, by hospital and management level. b) Percentage of managers completing training topics during their current management position by hospital and management level. **Appendix 3.** Difficulties experienced (percentage of managers) by hospital and management level

## Data Availability

The datasets used and/or analysed during the current study are available from the corresponding author on reasonable request.

## Abbreviations

CEO: Chief Executive Officer
ED: Executive Director
DED: Deputy Executive Director
HoA: Heads of the Administration and Functional Departments
DoA: Directors of Administration and Functional Departments
ADoA: Associate Directors of Administration and Functional Departments
HoCS: Heads of the Clinical Services
DoCS: Directors of Clinical Services
ADoCS: Associate Directors of Clinical Services
DoN: Directors of Nursing
QFSH: Qianfoshan Hospital
LCQH: Laichengqu Hospital
XXH: Xinxian Hospital
PG: Postgraduate
UG: Undergraduate
MCAP: Management Competency Assessment Partnership
PhD: Doctor of Philosophy
