# Supplementary material for: Developing senior hospital managers: does ‘one size fit all’? – evidence from the evolving Chinese health system
Source: BMC Health Serv Res. 2020 Apr 6;20:281. doi: 10.1186/s12913-020-05116-6 (PMC7137490; doi:10.1186/s12913-020-05116-6)
Supplement: Supplementary file 1 — Additional file 1: Appendix 1. MCAP Competency Likert Scale. Appendix 2. a) Percentage of managers completing training topics before taking up the current management position, by hospital and management level. b) Percentage of managers completing training topics during their current management position by hospital and management level. Appendix 3. Difficulties experienced (percentage of managers) by hospital and management level [file 12913_2020_5116_MOESM1_ESM.docx]

**Appendix 1. MCAP Competency Likert Scale**

|  | **Competency level** |
| --- | --- |
| 1 | May be capable of demonstrating minor aspects in my role |
| 2 | May be capable of demonstrating in my role, but not in all required aspects |
| 3 | Can fully demonstrate in my role with regular guidance |
| 4 | Can generally demonstrate in my role, but guidance is needed occasionally |
| 5 | Can demonstrate in my role independently without guidance |
| 6 | Always apply appropriately in my role with extensive experience |
| 7 | Always apply appropriately in my role, with extensive experience gained from diverse management roles at executive level |

**Appendix 2.**

**2a) Percentage of managers completing training topics before taking up the current management position, by hospital and management level**

| **Topic** | **QFSH** | | | **LCQH** | | | **XXH** | | | **Mean %** |
| --- | --- | --- | --- | --- | --- | --- | --- | --- | --- | --- |
|  | HoA | HoCS | DoN | HoA | HoCS | DoN | HoA | HoCS | DoN |  |
| **Conflict resolution** | **40** | **20** | **48*** | 14 | 24 | 32 | 18 | 7 | 8 | 26 |
| **Employee relations** | **40** | **26** | **47*** | 19 | 26 | 36 | 36 | 23 | 38 | 32 |
| **Safety training** | **47** | **31** | **48*** | 24 | 29 | 40 | 27 | 14 | 23 | 34 |
| **Performance management** | **43** | **32** | **51*** | 38 | 24 | 40 | 27 | 27 | 31 | 36 |
| **Leadership** | **30** | **22** | **48*** | 19 | 21 | 32 | 18 | 16 | 46 | 28 |
| **Human Resource Management** | **37** | **16** | **50*** | 19 | 17 | 28 | 27 | 14 | 31 | 26 |
| **Decision-making** | **37** | **20** | **38*** | 9 | 14 | 28 | 27 | 7 | 23 | 23 |
| **Health Information** | 23 | 17 | 17 | 9 | 17 | 24 | 0 | 11 | 15 | 17 |
| **Time management** | **33** | **20** | **52*** | 5 | 17 | 20 | 18 | 2 | 15 | 25 |
| **Communications** | **47** | **26** | **56*** | 38 | 31 | 52 | 36 | 29 | 54 | 37 |
| **Resource management** | **37** | **18** | **31*** | 9 | 12 | 28 | 36 | 14 | 15 | 22 |
| **Strategic planning** | 23 | 10 | 18 | 14 | 14 | 16 | 0 | 9 | 23 | 13 |
| **Planning & evaluation** | 17 | 13 | 18 | 5 | 12 | 16 | 9 | 4 | 0 | 13 |
| **Quality control** | 20 | 20 | 29 | 24 | 19 | 20 | 9 | 11 | 23 | 19 |
| **Values & ethics** | 10 | 14 | 19 | 9 | 17 | 16 | 9 | 14 | 15 | 15 |
| **Policy & Procedure** | 30 | 23 | 36 | 14 | 21 | 24 | 27 | 16 | 15 | 25 |
| **Mean percentage** | 32 | 20 | 38 | 17 | 20 | 28 | 20 | 14 | 23 | 24 |

QFSH: Jinan Qian FoShan Hospital; LCQH: Li Chang Qu Hospital; XXH: Xi Xian Hospital

HoA = Head of Administration and Functional Departments, HoCS= Head of Clinical Services, DoN = Directors of Nursing

* Bolded. Significant differences between management levels. Chi square=6.029 **―** 40.875; df=2; p=0.049̶̶̶ **―** <0.0005

**2b) Percentage of managers completing training topics during their current management position by hospital and management level**

| **Topic** | **QFSH** | | | **LCQH** | | | **XXH** | | | **Mean %** |
| --- | --- | --- | --- | --- | --- | --- | --- | --- | --- | --- |
|  | HoA | HoCS | DoN | HoA | HoCS | DoN | HoA | HoCS | DoN |  |
| **Conflict resolution** | **23** | **30** | **65*** | 33 | 29 | 40 | 18 | 18 | 23 | 36 |
| **Employee relations** | **27** | **25** | **70*** | 33 | 29 | 44 | 27 | 27 | 31 | 37 |
| **Safety training** | **43** | **41** | **73*** | 33 | 33 | 44 | 27 | 25 | 23 | 45 |
| **Performance management** | **53** | **44** | **79*** | 38 | 33 | 44 | 36 | 39 | 54 | 50 |
| **Leadership** | **30** | **31** | **75*** | 19 | 29 | 48 | 36 | 30 | 39 | 40 |
| **Human Resource Management** | **33** | **23** | **72*** | **19** | **19** | **48*** | 27 | 21 | 15 | 34 |
| **Decision-making** | **17** | **27** | **61*** | **14** | **12** | **36*** | 27 | 18 | 15 | 31 |
| **Health Information** | 17 | 23 | 30 | 19 | 14 | 20 | 0 | 11 | 15 | 21 |
| **Time management** | **17** | **20** | **75*** | 0 | 17 | 20 | 9 | 7 | 15 | 28 |
| **Communications** | **40** | **30** | **74*** | 48 | 36 | 52 | 18 | 41 | 46 | 43 |
| **Resource management** | **27** | **22** | **51*** | 9 | 17 | 32 | 9 | 16 | 23 | 27 |
| **Strategic planning** | **23** | **19** | **33*** | 14 | 12 | 24 | 0 | 16 | 0 | 20 |
| **Planning & evaluation** | 23 | 19 | 26 | 19 | 14 | 16 | 0 | 9 | 0 | 19 |
| **Quality control** | 27 | 29 | 41 | 14 | 19 | 12 | 9 | 27 | 39 | 29 |
| **Values & ethics** | 17 | 20 | 27 | 14 | 19 | 12 | 0 | 14 | 8 | 19 |
| **Policy & Procedure** | 37 | 39 | 44 | 19 | 21 | 20 | 27 | 25 | 23 | 35 |
| **Mean percentage** | **28** | **28** | **56*** | 22 | 22 | 32 | 17 | 21 | 23 | 32 |

QFSH: Jinan Qian FoShan Hospital; LCQH: Li Chang Qu Hospital; XXH: Xi Xian Hospital

HoA = Head of Administration and Functional Departments, HoCS= Head of Clinical Services, DoN = Directors of Nursing

* Bolded. Significant differences between management levels. Chi square=7.928**―**96.407; df=2; p = 0.019**―**<0.0005

**Appendix 3.**

**Difficulties experienced (percentage of managers) by hospital and management level**

| **Difficulty type** | **QFSH** | | | **LCQH** | | | **XXH** | | | **Mean %** |
| --- | --- | --- | --- | --- | --- | --- | --- | --- | --- | --- |
|  | HoA | HoCS | DoN | HoA | HoCS | DoN | HoA | HoCS | DoN |  |
| **Peer conflict** | **57** | **28** | **40*** | **48** | **24** | **16*** | 18 | 23 | 8 | 31 |
| **Team conflict** | **33** | **26** | **45*** | 19 | 21 | 16 | 0 | 18 | 23 | 28 |
| **Staff turnover** | **33** | **4** | **8*** | 14 | 21 | 20 | 9 | 21 | 23 | 11 |
| **Patient conflict** | **3** | **48** | **59*** | 38 | 45 | 36 | 36 | 39 | 62 | 46 |
| **Innovative teamwork** | **30** | **39** | **54*** | 10 | 21 | 12 | 18 | 14 | 15 | 34 |
| **Staff hiring** | **38** | **8** | **4*** | 10 | 4 | 0 | 9 | 5 | 0 | 7 |
| **Loss of skilled staff** | 10 | 10 | 13 | 19 | 17 | 16 | 9 | 16 | 15 | 12 |
| **Team skill building** | 13 | 25 | 25 | 14 | 7 | 8 | 0 | 14 | 15 | 20 |
| **Unethical problems** | 7 | 11 | 12 | 5 | 5 | 4 | 0 | 2 | 0 | 8 |
| **Supervisor confrontation** | 13 | 9 | 12 | 5 | 2 | 4 | 0 | 11 | 8 | 9 |
| **Employee performance** | **17** | **39** | **53*** | 33 | 33 | 20 | **0** | **27** | **62*** | 38 |
| **Decision-making & change** | 37 | 30 | 39 | 14 | 17 | 4 | 9 | 30 | 39 | 29 |
| **New skill acquisition** | 23 | 32 | 37 | 38 | 43 | 36 | 27 | 18 | 31 | 33 |
| **Expected work quality** | 37 | 32 | 39 | **24** | **41** | **12*** | 18 | 14 | 23 | 31 |
| **Management outcomes expectations** | 30 | 26 | 36 | 14 | 26 | 12 | 9 | 16 | 23 | 26 |
| **Mean percentage** | 25 | 24 | 32 | 20 | 22 | 14 | 11 | 18 | 23 | 24 |

QFSH: Jinan Qian FoShan Hospital; LCQH: Li Chang Qu Hospital; XXH: Xi Xian Hospital

HoA = Head of Administration and Functional Departments, HoCS= Head of Clinical Services, DoN = Directors of Nursing

* Bolded. Significant differences between management levels. Chi square=6.239 **―** 30.402; df=2; p=0.044 **―** <0.0005
